# Marsupial Gut Microbiome

**DOI:** 10.3389/fmicb.2020.01058

**Published:** 2020-05-29

**Authors:** Rowena Chong, Yuanyuan Cheng, Carolyn J. Hogg, Katherine Belov

**Affiliations:** School of Life and Environmental Sciences, The University of Sydney, Sydney, NSW, Australia

**Keywords:** gut microbiome, wildlife conservation, marsupial, captivity, translocation, dysbiosis

## Abstract

The study of the gut microbiome in threatened wildlife species has enormous potential to improve conservation efforts and gain insights into host-microbe coevolution. Threatened species are often housed in captivity, and during this process undergo considerable changes to their gut microbiome. Studying the gut microbiome of captive animals therefore allows identification of dysbiosis and opportunities for improving management practices in captivity and for subsequent translocations. Manipulation of the gut microbiome through methods such as fecal transplant may offer an innovative means of restoring dysbiotic microbiomes in threatened species to provide health benefits. Finally, characterization of the gut microbiome (including the viral components, or virome) provides important baseline health information and may lead to discovery of significant microbial pathogens. Here we summarize our current understanding of microbiomes in Australian marsupial species.

## Introduction

The gut microbiome plays an important role in many physiological processes including nutrition (Kau et al., 2011), immunity (Round and Mazmanian, 2009), metabolism (Musso et al., 2011), brain functions and behavior (Rogers G. et al., 2016). In humans, the highly diverse gut bacterial communities have been found to play a wide range of symbiotic functions that are essential for maintaining the health of the host, and disturbances to the gut microbiome structure have been associated with various diseases, such as diabetes, inflammations, metabolic or autoimmune disorders, infections, and cancer (reviewed in Kho and Lal, 2018). Certain attributes of the gut microbiome have been implicated in an increased risk for an individual to develop certain diseases, such as a high Firmicutes:Bacteroidetes ratio in obesity (Ley et al., 2006), and Enterobacterial blooms in inflammatory diseases of the gut (Zeng et al., 2017). Advances in sequencing technologies in recent years have also allowed the development of new methods for studying the gut virome, another important component of the gut microbial ecosystem, revealing a high richness of gut viral community and various potential beneficial functions of viruses (e.g., bacteriophages) in mediating host microbiome adaptation and stability (Ogilvie and Jones, 2015).

Much of what we know about the gut microbiome so far stems from studies in humans or animal model species, but recent studies have increasingly focused on wildlife biology and conservation (Trevelline et al., 2019). These offer a wealth of knowledge about the abundance and diversity of microbes that inhabit wildlife species across diverse taxa, including the diverse lineage of marsupials. Australian marsupials represent a unique evolutionary lineage of mammals that has dominated the Australian continent. A long history of geographical isolation has led to the diversification of marsupial species in terms of their biology, diets and life history traits (Nipperess, 2015). Here we will review our current understanding of the gut microbiome of marsupials and how this knowledge can be applied to further our understanding of marsupial health, host-microbiome coevolution and conservation.

## Baseline Characterization of Marsupial Gut Microbiome

### Tasmanian Devil

The Tasmanian devil (*Sarcophilus harrisii*; “devil” hereinafter) is the world’s largest living carnivorous marsupial from the family Dasyuridae. Once widespread throughout Australia, it became extinct on the mainland about 400 to 3,000 years ago (Archer and Baynes, 1972; Brown, 2006) and is now endemic to the island state of Tasmania. Modern devils are facing extinction due to a fatal contagious cancer called devil facial tumour diseases (DFTD) (Pemberton, 2019). Since its discovery in 1996, DFTD has spread over 75% of the state and caused declines of up to 80% of wild devil populations (Lazenby et al., 2018). This has resulted in devils being listed as Endangered by the IUCN (International Union for Conservation of Nature) and under the Environment Protection and Biodiversity Conservation (EPBC) Act (Australia). A large amount of effort has gone into furthering our understanding of devil biology to facilitate conservation efforts, including population genetics (Jones et al., 2004; Miller et al., 2011) and the etiology of DFTD (Pearse and Swift, 2006; Pye et al., 2016). Tasmanian devils are predominantly scavengers, but are also known to hunt, consuming a wide range of prey items from marcopods to insects, birds and fish (Table 1).

**TABLE 1:** Comparison of diet, habitat, and gut microbiome in marsupials.

Species	Feeding strategy	Diet	Distribution and habitat	Major gut bacteria^a^	Firmicutes: Bacteroidetes^b^	References
Tasmanian devil	Generalist carnivore	Mammals, insects, birds, fish, and carrion	Tasmania. Inter-tidal to sub-alpine; predominantly with sclerophyll forests. Mosaic landscape of forest and farmland.	• Firmicutes 53.5 ± 3.9% • Proteobacteria 18.6 ± 3.5% • Fusobacteria 13.8 ± 4.5% • The most abundant genus: *Clostridium*	45:1	Pemberton et al., 2008; Cheng et al., 2015; Rogers T. et al., 2016; Pemberton, 2019
Northern quoll	Generalist omnivore	Mammals, birds, reptiles, frogs, invertebrates, fruit, and carrion	Northern Australia. Arid and coastal zones; inland to approximately 200 km from coast. Tropical lowland savanna.	• Firmicutes 58.1 ± 21.3% • Proteobacteria 34.4 ± 21.3% • The most abundant genus: *Enterococcus*	13:1	Oakwood, 2000; Hernandez-Santin et al., 2016; Dunlop et al., 2017; Burke et al., 2018
Koala	Specialist folivore	Eucalyptus foliage (different populations feed on different types of *Eucalyptus*)	Eastern to Southern Australia Eucalypt forest and woodland communities.	• Firmicutes 45% • Bacteroidetes 23% • Proteobacteria 15% • The most abundant genus: *Bacteroides*	2:1	Cork et al., 1983; Moore et al., 2005; Shiffman et al., 2017; Johnson et al., 2018
Common wombat	Generalist herbivore	Grass and snow grass	Tasmania and south-eastern Australia. Any elevation in south of their range; in mountainous areas in QLD. Rainforest, eucalyptus forest, woodland, alpine grassland, and coastal areas.	• Firmicutes 61% • Bacteroidetes 18% • The most abundant genus: *Bacteroides*	3.4:1	Rishworth et al., 1995; Groves, 2005; Evans et al., 2006; Shiffman et al., 2017
Macropods (*Macropus giganteus*, *Macropus rufus*, and *Macropus robustus*)	Generalist herbivores	Various grass and herbaceous plant species	A wide range of habitats across Australia, ranging from arid desert zones to temperate forests, and alpine regions.	• Bacteroidetes 48.3 ± 9.2% (mostly Prevotellaceae) • Firmicutes 47.3 ± 9.9% (mostly Lachnospiraceae)	1:1	Jarman, 1984; Gulino et al., 2013

**^*a*^Tasmanian devil, koala, and wombat data was collected using fecal samples; the quoll and macropod studies used cloacal swabs and foregut fluid, respectively. ^*b*^Ratios were estimated as (average relative abundance of Firmicutes)/(average relative abundance of Bacteroidetes); individual level F:B ratios may vary greatly.**

**FIGURE 1:**
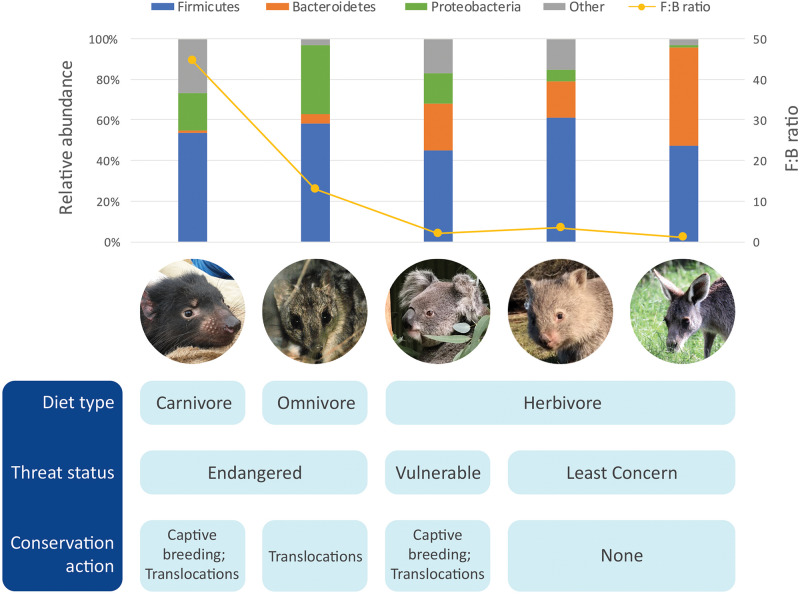
Comparison of gut microbiome of five marsupials [data from Barker et al. (2013), Gulino et al. (2013), Cheng et al. (2015), Shiffman et al. (2017), Burke et al. (2018)].

More recently, the microbiome of the devil also became the focus of research. Initial microbiome characterization on the devils using 16S rRNA gene amplicon sequencing of the V1–V3 region generated baseline information of the bacterial communities in the gut (feces), pouch, skin and oral cavity (Cheng et al., 2015). Across all body sites, bacterial phyla Firmicutes, Proteobacteria, Fusobacteria, Bacteroidetes, and Actinobacteria were the top five constituents. However, compared to the other three microbiome types, the gut microbiome had significantly higher phylotype richness (Cheng et al., 2015). The most abundant bacterial phyla found within the devil gut microbiome was Firmicutes (53.5 ± 3.9%), followed by Proteobacteria (18.6 ± 3.5%), and fusobacteria (13.8 ± 4.5%) (Figure 1 and Table 1; Cheng et al., 2015). *Clostridium*, a bacterial genus known to contain species with protein decomposition and amino acid degradation activities (Fonknechten et al., 2010), was identified as the most common bacteria in devil gut flora (18.5 ± 2.4%), which speculatively could be an indication of the gut flora having evolved to adapt to the host’s carnivorous feeding strategy. The level of Proteobacteria (primarily Gammaproteobacteria and Alphaproteobacteria) observed in devil gut microbiome is relatively higher than that found in many other mammalian species (on average 8.8% in mammals based on Ley et al., 2008). Particularly Enterobacteriaceae, a family of Gammaproteobacteria, accounts for approximately 9.4% of the devil gut flora. This bacterial family is known to contain many symbionts such as *Escherichia coli*, *Klebsiella* spp., and *Proteus* spp., and in humans, dysbiosis involving Enterobacteriaceae have been associated with various inflammatory gut diseases (Zeng et al., 2017).

Another important feature of the devil gut microbiome is the low prevalence of Bacteroidetes (1.2 ± 0.6%), which leads to a high Firmicutes to Bacteroidetes ratio (*F*:*B* ratio; approximately 45:1 in devils) (Cheng et al., 2015). It has been found in humans and mice that a high *F*:*B* ratio (the “obese microbiome”) is associated with high efficiency in energy harvest from the diet and an increased risk for the host to develop obesity, while the increase of Bacteroidetes and decrease of Firmicutes can lead to weight loss (Ley et al., 2006; Turnbaugh et al., 2006). Interestingly, low levels of Bacteroidetes have also been observed in the gut microbiome of many other carnivorous mammals besides devils, including the cheetah (*Acinonyx jubatus*) (Menke et al., 2017), spotted hyena (*Crocuta crocuta*), polar bear (*Ursus maritimus*) (Ley et al., 2008), and northern quoll (*Dasyurus hallucatus*; further discussed below). These findings suggest that a high *F*:*B* ratio could be a feature of carnivorous species which is possibly related to the need to efficiently harvest and store energy from limited food sources (Cheng et al., 2015). In the devil, this feature is also in line with their feeding habit, whereby they typically gorge up to 40% of their body weight in a single meal, followed by several days of no feeding (Pemberton and Renouf, 1993).

In addition to the gut bacterial microbiome, a recent study reported the characterization of devil fecal virome and the identification of 24 novel marsupial-associated viruses as well as known mammalian pathogens such as rabbit haemorrhagic disease virus (Chong et al., 2019b). Some notable marsupial-associated viruses identified include astroviruses, picobirnaviruses, parvoviruses, papillomaviruses, polyomaviruses and a gammaherpesvirus. Among these, picobirnaviruses have recently been found to possess bacteriophage properties (Krishnamurthy and Wang, 2018) and thus can potentially play a role in the regulation of gut bacterial community and protection against pathogenic bacteria (Mukhopadhya et al., 2019). Prior to this study, only a single gammaherpesvirus affecting both captive and wild devils has been recorded in the literature (Stalder et al., 2015), demonstrating a significant lack of knowledge in this area. Although much is still unknown regarding what roles the viruses identified in devil gut flora may play on host health, the viral sequences isolated through devil gut virome characterization provide a useful resource for future research toward illuminating activities and functions of mammalian gut viruses. Further investigations of gut virome in more marsupial species will be needed to understand the structure and function of viruses in the gut microenvironment of marsupials.

### Northern Quoll

The northern quoll (*D. hallucatus*) is an omnivorous marsupial from the family Dasyuridae. Found predominantly in the northern regions of Australia, they are currently listed as endangered and are found distributed in fragmented areas across northern Australia (Braithwaite and Griffiths, 1994). Northern quolls are generalists consuming a wide prey base including vertebrate and invertebrate prey and fruit (Table 1). Using cloacal swab as a non-invasive proxy for the gut, the gut microbiome of the northern quoll was characterized using 16S rRNA amplicon sequencing of the V3–V4 region (Burke et al., 2018). Similar to its close carnivorous relative, the Tasmanian devil, the northern quoll cloacal microbiome shows high abundance of Firmicutes (58.1 ± 21.3%) and Proteobacteria (34.4 ± 21.3%) and low abundance of Bacteroidetes (4.5 ± 13.85%) (Figure 1; Burke et al., 2018). In addition, the northern quoll gut microbiome was characterized by a high abundance of Enterococcus (27.3 ± 22.4%) compared to other mammalian species (∼1% in humans) (Dubin and Pamer, 2014). The similarities between the northern quoll and devil gut microbiome in the higher taxonomic levels can possibly be attributed to their close phylogenetic relationship, as well as similar carnivorous diets. However, it should be noted that due to different sampling methods that have been used for the two species (feces vs. cloacal swab), the results from the two studies on devils and quolls may not be directly comparable.

### Koala

The koala (*Phascolarctos cinereus*) is an arboreal folivore endemic to Australia and the last surviving member of the family Phascolarctidae. Koalas occur across eastern Australia in a wide range of habitat types (Table 1). Yet they are a dietary specialist, feeding solely on the foliage from species of *Eucalyptus* (Cork and Sanson, 1991). Various anatomical and physiological adaptations enable the koalas to survive on a diet that is low in proteins and high in lignified fiber and phenolic compounds that would make it toxic to other animals. The hindgut, including the caecum and proximal colon of a koala is significantly enlarged (Cork and Sanson, 1991), making it one of the largest in any known mammals (Krockenberger and Hume, 2007). The mean retention times of solutes and larger particles of digesta in the digestive tract in koalas are both longer than have been reported in most other mammals, including many other eucalypt-specialist marsupial folivores (Krockenberger and Hume, 2007), allowing the potential for relatively extensive microbial degradation and nutrient extraction from the nutritionally poor foliage. In addition, endogenous enzymes produced in the liver have also been found to assist the koala in coping with toxic plant secondary metabolites (PSMs) in their *Eucalyptus* diets (Ngo et al., 2000). As with other herbivores, the koala relies on microbes in their gut for digestion of plant material through hydrolysis and fermentation.

Characterization of the koala hindgut microbiome revealed a dominance of Firmicutes and Bacteroidetes, consistent with many other species (Figure 1; Barker et al., 2013). The *F*:*B* ratio varies significantly across the hindgut, with a low ratio close to 1 (1.3:1) found in the caecum, and significantly higher ratios of 6:1 and 3:1 in the colon and fecal pellet, respectively, suggesting differential microbial fermentation processes taking place at various sites (Barker et al., 2013). Due to its unusual diet, a number of studies have focused on elucidating the gut microbiome’s contribution to the host’s ability to digest and detoxify Eucalyptus. Early investigations using culture-based techniques identified presence of tannin degrading microorganisms across the koala’s gastrointestinal tract, including *Streptococcus gallolyticus* and *Lonepinella koalarum* from the Pasteurellaceae family (Osawa, 1990; Osawa et al., 1995). Furthermore, comparative metagenomics analysis of the gut microbiome between koala and its closest living relative, the wombat has enabled identification of other key microbial linages and functional pathways unique to the koala. Several microbial lineages thought to play conserved roles in fiber degradation and urea recycling, both of which are essential metabolic pathways for herbivorous species, were found in both the koala and wombat (Shiffman et al., 2017). For example, fibrolytic bacteria from the genus *Bacteroides* and *Ruminococcus* were found consistently across all koala and wombat samples. These fibrolytic bacteria metabolize complex plant compounds into short-chain fatty acids, which can then be easily absorbed by the host (Barboza and Hume, 1992). Urease-containing *Succinivibrionaceae* bacterium found in both species are thought to assist in urea degradation. In mammals, ammonia, the toxic end-product of protein catabolism, is converted into urea through the urea cycle for elimination; it is estimated that approximately 20% of urea is degraded by urease-expressing gut bacteria through the gastrointestinal tract, with the remaining eliminated through renal excretion (Ramezani et al., 2016). One important distinction between koala and wombat gut microbiome is that members of the family *Synergistaceae* were detected at relatively high abundance (>4–17%) in the koala but absent in wombat. These bacterial populations are predicted to encode multiple pathways related to the degradation of toxic *Eucalyptus* plant secondary metabolites (PSMs), therefore playing a key role in the koala’s ability to survive in a specialized dietary niche (Shiffman et al., 2017).

### Wombat

The wombat is the koala’s closest living relative, both belonging to the suborder Vombatiformes. The family Vombatidae consists of three species, the common wombat (*Vombatus ursinus*), the southern-hairy nosed wombat (*Lasiorhinus latifrons*) and the northern-hairy nosed wombat (*Lasiorhinus krefftii*). The common wombat is found across a range of habitats in Tasmania and south-eastern Australia (Table 1), with the southern-hairy nosed wombat found in southern Australia, and the northern-hairy nosed wombat isolated to Queensland. Unlike the koala, wombats are a generalist herbivore that primarily grazes on grass (Rishworth et al., 1995).

Characterization of the gut microbiome has been carried out in two species of wombats, the southern hairy-nosed wombat and common wombat. Based on 16S rRNA amplicon sequencing of the V6 to V8 region, there was a dominance of Firmicutes (∼61%) and Bacteroidetes (∼18%) and relatively low *F*:*B* ratio (3.4:1) (Figure 1; Shiffman et al., 2017). Compared to the koala, higher levels of xylanases were found (7.6% vs. 1.9% in the koala), which could be attributed to the higher content of hemicellulose in the wombat diet (Rishworth et al., 1995; Hume, 1999). Several distinct microbes were also only detected in the wombat, including unclassified members of the family *Christensenellacea*, the order *Clostridiales*, and the genus *Ruminococcus* (Shiffman et al., 2017).

### Macropods

This family of Macropodidae, including species of kangaroos and wallabies, is found in a wide range of habitats across Australia, ranging from arid desert zones to temperate forests and alpine regions. They are grazing generalist herbivores, foraging on a range of grass and herbaceous plant species depending on their environment (Jarman, 1984). All species of macropods are foregut fermenters (Hume, 1999). Consequently, the gastrointestinal tracts of macropods are generally characterized by an enlarged forestomach, sacciform, and tubiform where microbial fermentation of plant material takes place (Hume, 1999). Early studies based on 16S rRNA amplicon sequencing of the V3–V4 region again identified Bacteroidetes and Firmicutes as key constituents of macropod (*Macropus giganteus*, *Macropus rufus*, and *Macropus robustus*) foregut microbiome (48.3 ± 9.19% and 47.3 ± 9.85%, respectively) (Figure 1; Gulino et al., 2013). A number of OTUs identified in the macropod foregut microbiome shared highly percentage of homology to known fibrolytic bacteria such as *Ruminococcus flavefaciens* and *Butyrivibrio fibrisolvens*, which were identified as key microbes responsible for fibrolytic digestion (Hespell et al., 1987; Miller et al., 2009). In the tammar wallaby (*Macropus eugenii*), it has been reported that pouch young (40 and 56 days old) have a gut flora dominated by Firmicutes and Actinobacteria that is distinct to the maternal pouch and oral microbiome, highlighting the possibility of the gut microbiota of marsupial pouch young arising from the maternal milk (Chhour et al., 2010). However, it should be noted that this early study used a low throughput cloning-based method for sequencing 16S rRNA genes, which may not have the power to fully reveal the complexity and comprehensive structure of the microbiomes surveyed.

## Implications for Marsupial Biology and Conservation

### Dysbiosis in Captivity and Implications for Translocation/Reintroduction

A commonly used tool in conservation management is captive breeding for those species which are suffering significant population declines (Harley et al., 2018). Yet life in captivity can present a range of extreme lifestyle changes, many of which may affect the host microbiome. A growing number of studies have focused on determining the effects of captivity on wildlife microbiomes, with many providing evidence of microbiome perturbations (Amato, 2013; Kohl et al., 2014; McKenzie et al., 2017). Significant differences in the gut microbiome composition of captive animals relative to their wild counterparts have been frequently observed in many species. This is particularly apparent in carnivorous and omnivorous species, where the supply of natural and diverse diets in an artificial setting is often restricted (Nakamura et al., 2011; Guan et al., 2016). In Tasmanian devils, evidence of microbiome dysbiosis has been detected, where captive individuals showed significantly different gut microbiome compositions and lower microbial diversity compared to their wild counterparts (Cheng et al., 2015). Interestingly, the type of captive enclosure influenced gut microbiome composition and diversity in the devils. Of the two types of captive enclosures studied, devils that were housed in more intensive, zoo-based facilities had lower microbial diversity in their gut than those housed in larger, group housing enclosures. Those that are housed in group enclosures also have gut microbiomes that more closely resemble the microbiome of wild devils, suggesting free-range or group enclosures to be a more preferable housing option for managing devil microbiomes in captivity (Cheng et al., 2015). Currently the impact of a depauperate microbiome on devils remains unclear, but it has been suggested that the low diversity of gut microbiome in captive devils may lead to an increased risk of obesity (Le Chatelier et al., 2013), which can consequently cause reduced success rate of captive breeding (Cheng et al., 2015).

In contrast, it has been reported that captivity does not appear to significantly alter the gut microbiome in koalas, as both captive and wild koalas share very similar and consistent microbiome compositions at the phyla and genus level (Alfano et al., 2015). The lack of differentiation between captive and wild microbiomes has mostly been observed in herbivorous species, such as even-toed ungulates (bovids and giraffes) (McKenzie et al., 2017). Alterations to the microbiome in captivity can be driven by many factors, including changes to the natural diets (Clayton et al., 2016), reduced environmental microbial reservoirs (Loudon et al., 2014), cohabitation with other species (Lemieux-Labonté et al., 2016), and antibiotic use (Plummer et al., 2005; Dahlhausen et al., 2018). The strict dietary requirements of koalas mean that captive and wild individuals likely feed on similar *Eucalyptus* diets, which could have resulted in limited differentiations in their gut microbiome. One limitation though with the study by Alfano et al. (2015) is the small sample size consisting of only two captive koalas (plus data of two wild koalas from an earlier study). Further studies using a larger sample size will be needed to verify the hypothesis that captive and wild koalas have similar gut microbiomes.

An important aspect of many captive breeding programs is the reintroduction of animals back into the wild (Seddon et al., 2007). Microbiome perturbations observed in captivity may underlie poor host health, which may in turn impact the reintroduction success and post-release survival of captive individuals (Redford et al., 2012). For example, increased abundance of pathogenic microbes and disease-associated pathways in the captive cheetah gut microbiome may explain the poor reproductive rates and high prevalence of bacterial infections associated mortality (Menke et al., 2017). Similarly in the grouse (Tetrao urogallus), microbiome disturbances (Wienemann et al., 2011) as well as anatomical changes, such as shorter small intestines and caeca observed in captivity (Liukkonen-Anttila et al., 2000), may compromise digestion likely leading to high mortality of captive birds upon release to the wild (Seiler et al., 2000). With concerns about the consequences of dysbiosis in captivity, and the potential implications for the reintroduction of captive devils back into the wild, the gut microbiomes of translocated devils were monitored for temporal changes over the course of translocation to understand how translocation may influence devil gut microbiome (Chong et al., 2019a). Comparisons between the microbiome of released devils before and after translocation showed significant shifts in composition and diversity, and that released devils began to re-acquire the wild, incumbent microbiome as early as 3–4 weeks post-release (Chong et al., 2019a). This result suggests that microbiome perturbations as a result of captivity in a carnivorous species, such as the devils, are not necessarily permanent. Studies investigating changes in the gut microbiome post-release are scarce but can provide important insights into the impact of translocation on the host-associated microbiome, allowing evaluation and improvement of translocation success.

### Gut Microbiome Management in Wildlife Conservation

Bioaugmentation of the microbiome through probiotic therapy or fecal microbiome transplantation is a new and emerging field in microbiome research, especially within the context of wildlife conservation (McKenzie et al., 2018; Trevelline et al., 2019). Augmenting or manipulating the microbiome may provide numerous benefits, such as restoring dysbiotic microbiome for improved physiological functions and animal health (Kueneman et al., 2016), and mitigating disease risk (Bletz et al., 2013). In the wild, koalas often have access to different *Eucalyptus* spp. but majority feed exclusively on a specific food tree (Brice et al., 2019). The koala gut microbiome has been suggested to play a role in their dietary preferences. For example, the gut microbiome of koalas that preferentially feed on messmate gum (*Eucalyptus obliqua*) have higher abundances of fibrolytic bacteria and are more adapted to using different complex carbohydrate sources than those feeding on manna gum (*Eucalyptus viminalis*), one of the main food trees for koalas in many areas (Brice et al., 2019). Microbial richness and diversity were also found to be lower in the microbiome of koalas feeding on manna gum, likely to be linked to a greater energy harvest from this species of *Eucalyptus* compared to messmate (Brice et al., 2019). Therefore it has been hypothesized that the inability of the majority of koalas to shift diets to messmate is due to a lack appropriate gut microbial assemblage for optimal digestion (Blyton et al., 2019). With the continuous threat of habitat loss and limited resources, the ability to shift diet and utilize different food sources is crucial for the survival and persistence of koalas in the wild. In a study by Blyton et al. (2019), wild koalas that previously fed on manna gum were inoculated with faecally derived microbes from koalas that feed on messmate. Although on average, the treatment koalas did not show a significant increase in their messmate consumption after inoculation, their gut microbiome shifted significantly to resemble the microbiomes of koalas that feed primarily on messmate. Also importantly, a pattern was observed that koalas showing a more prominent shift in the gut flora consumed more messmate. As such, fecal transplant between koalas feeding on different Eucalyptus species may be useful in introducing beneficial microbes to the gut microbiome that will enable koalas to adapt to and utilize more variety of food sources. This may prove to be particularly important when translocating koalas to areas with different *Eucalyptus* tree species. Meanwhile, it also needs to be emphasized that further research will be needed to evaluate the broader impact and safety (e.g., potential disease transmission) of such treatments in wild species.

### Microbiome in Health and Disease

Infectious diseases are major threats to wildlife species. In marsupials, a well-known example is *Chlamydia* infections in koalas. Infections caused by *Chlamydia pecorum* and *Chlamydia pneumoniae* can cause conjunctivitis, blindness, pneumonia, urinary tract and reproductive tract infections, and infertility (Brown et al., 1987). Antibiotic treatments are routinely used in wildlife hospitals to treat infections, but have been suggested to cause disruptions to the normal intestinal microbial communities, resulting in adverse side effects (Polkinghorne et al., 2013; Dahlhausen et al., 2018). Results from a study by Dahlhausen et al. (2018) found that koalas that were treated with antibiotics for *chlamydia* and subsequently died had lower microbial diversity and abundance of tannin-degrading bacteria, *Lonepinella koalarum*, in their gut than koalas recovered after treatment. Although the study did not detect a significant difference in the gut bacterial richness between antibiotic-treated koalas and control individuals, possibly at least partly due to the limited number of controls (two koalas), the comparison of microbiome between pre-treatment and post-treatment samples revealed that antibiotic treatments may influence the composition and adaptation of gut microbiome of koalas and affect the abundance of beneficial microbes with functions (such as detoxification of *Eucalyptus*) essential to the health and survival of the species.

With increasing usage of antibiotics in wildlife medicine, antibiotic resistance is of growing concern for the health and conservation of threatened species (West et al., 2019). Evidence of antibiotic resistance has been detected in a number of wildlife species including Iberian lynx (*Lynx pardinus*) (Sousa et al., 2014) and the Australian sea lions (*Neophoca cinerea*) (Delport et al., 2015). In marsupials, bacterial genetic elements associated with antibiotic resistance genes (class 1 integrons) have also been found in the gut microbiome of the endangered brush-tailed rock-wallabies (*Petrogale penicillata*) living in captivity (Power et al., 2013). This raises concerns about the future effectiveness of antibiotic treatments, as well as the potential spread of resistance into wild populations through the translocation of these captive individuals. Careful use of antibiotic treatment, as well as continuous efforts to develop antibiotic alternatives, are paramount to prevent the rise of antibiotic-resistant diseases in threatened wildlife.

Another emerging field of research in wildlife gut microbiome is the study of the gut virome. So far, the overall knowledge on functions of gut virome is still quite limited even in model species. Most of the current understanding on the potential beneficial effect of gut viruses surrounds bacteriophages, which have been suggested to play a part in regulating and maintaining the balance of bacterial community (Ogilvie and Jones, 2015). Emerging evidence also suggests that gut viruses interact with the host immune system and are likely sources of immune variation (Neil and Cadwell, 2018). The Tasmanian devil was the first marsupial species in which the gut viral communities have been characterized in great depth (Chong et al., 2019b). Identification of viruses, some of which are potentially pathogenic is important for understanding and safeguarding devil health. Further work is required to elucidate the pathogenicity of novel viruses. The use of a metagenomics approach to categorize the viral components of the gut microbiome in marsupial is still in its infancy but has enormous coding potential. Current knowledge on the diversity of viruses found in marsupials is scarce and virome studies will provide important baseline health information, as well as insights into host-microbe interactions and the phylogenetic history of viruses infecting this evolutionary unique group of mammals.

## Conclusion

Australia has one of the highest extinction rates of mammals in the world (Woinarski et al., 2015). Conservation biologists are constantly searching for ways to protect threatened wildlife species from extinction. With advances in sequencing technology, our ability to catalog and study the complex host-associated gut microbiome has improved substantially in recent years. Consequently, there has been a paradigm shift focusing on understanding the importance of the gut microbiome in threatened wildlife species and how the knowledge gained can contribute to conservation efforts. In this review, we have provided numerous examples of how studying the gut microbiome has advanced our understanding of marsupial biology (such as the complex microbial digestion of toxic *Eucalyptus* in koalas), as well as how to facilitate conservation through managing the microbiome in captive populations and during translocations. In addition, the ability to manipulate the gut microbiome through methods such as fecal inoculations proves to be an exciting avenue for future research in wildlife health. For many marsupial species, baseline characterization of their gut microbiome is still required. This will be an essential first step in understanding the overall patterns of microbial composition and diversity, thus providing a springboard for studying dysbiosis, particularly in relation to multiple anthropogenic pressures and environmental changes, such as captive management and habitat disturbances.

## Author Contributions

RC wrote the manuscript with input from KB. YC and CH made Figure 1 and Table 1 and carried out major revisions of the manuscript.

## Conflict of Interest

The authors declare that the research was conducted in the absence of any commercial or financial relationships that could be construed as a potential conflict of interest.
